# Editorial: Innate immune responses to SARS-CoV-2 in infected and vaccinated individuals

**DOI:** 10.3389/fimmu.2023.1141405

**Published:** 2023-02-08

**Authors:** Nitin K. Saksena, Pedro A. Reche, Srinivasa Reddy Bonam, Nicasio Mancini

**Affiliations:** ^1^ Institute for health and Sports (IHES), Victoria University, Melbourne, VIC, Australia; ^2^ Research & Development (R&D), Aegros Therapeutics, Sydney, NSW, Australia; ^3^ Department of Immunology, School of Medicine, Complutense University of Madrid, Madrid, Spain; ^4^ Department of Microbiology and Immunology, University of Texas Medical Branch, Galveston, TX, United States; ^5^ Laboratory of Medical Microbiology and Virology, University Vita-Salute San Raffaele, IRCCS San Raffaele Hospital, Milan, Italy

**Keywords:** SARS-CoV-2, acute respiratory disease, vaccines, mRNA vaccines, neutralization, viral variants

The COVID-19 pandemic triggered the intensity of the pursuit of vaccines that had not been seen before and were developed at an unprecedented speed, approved, and delivered for human use globally. During the pandemic, the first mRNA vaccine, a new paradigm in vaccinology for SARS-CoV-2, was also developed by Pfizer-BioNTech and Moderna. Most current vaccines in use have aimed to induce specific adaptive immunity by taking advantage of host T-cell responses and virus-neutralizing antibodies (nAbs). Innate immunity, critical to host defense against infections, is triggered by a family of pattern recognition receptors, which in turn induce interferons and cytokines, activating myeloid and lymphoid cells to provide immune protection against a range of viral and bacterial infections. Through thematic convergence, the assemblage of 18 original articles and reviews presented in this special issue, entitled “Innate immune responses to SARS-CoV-2 in infected and vaccinated individuals,” discusses various perspectives and provides a profound and multidisciplinary understanding of SARS-CoV-2 vaccines and innate immune responses. This information provided in the special issue is critical to defining the limitations of current approaches and facilitating greater understanding and refinement of current and future vaccine design.

While the COVID-19-approved vaccines are generally safe, serious side effects have remained an issue with both mRNA and non-mRNA vaccines, and more time is needed to assess their potential long-term effects. The limited production of heterogeneous neutralizing antibodies and their short durability *in vivo* remain unsolved and need to be addressed. Nonetheless, even though the current COVID-19 vaccines have not always been used in concert with the circulating strain, they have been shown to protect against severe disease, which is usually typified by pneumonia, cytokine storm, acute respiratory distress syndrome (ARDS), multiorgan failure, and death. Most current vaccines also lack variant-specificity; therefore, the newly emerging viral variants have been seen to breach host immunity through mutations during the COVID-19 pandemic. As a result, we have seen millions of re-infections globally post-vaccination and post-booster vaccination, with varying recovery rates depending on the host immunology and circulating mutations in the viral strains (Kumar et al.). For instance, patients infected with the Delta variant of SARS-CoV-2 were found to have a slower recovery rate than their counterparts infected with Alpha or Omicron variants. This trend was consistent in both vaccinated and unvaccinated patients. Omicron causes less severe disease than Delta, which could be attributed to Omicron being less able to infect the lungs as it does in the upper airways, thereby resulting in less severe disease than Delta, coupled with a lower risk of hospitalization and ICU admissions (40–80% and lower than that of Delta) and death (60% less death rate than Delta) (1). Möhlendick et al determined that the risk of developing an Omicron breakthrough infection was independent of vaccination scheme sex, body mass index, smoking status or pre-existing conditions, but it correlated with lower antibody responses induced after booster immunization.

Overall, such observations are significant in defining vaccine efficacy, as viral mutations that define viral infectivity and transmissibility work together to evade the host-and vaccine-induced immunity, and require further investigation to create specific and durable SARS-CoV-2 vaccines. Furthermore, unless SARS-CoV-2 vaccines can provide long-term sterilizing immunity, they will most likely become seasonal and will require yearly immunizations, as is known for the influenza virus. To develop a vaccine of this kind, a clear understanding of the mechanism of interaction of the virus with the host and the host’s response to the vaccine is urgently needed. More insights are needed into the innate immune system components, including monocytes, macrophages, dendritic cells, and granulocytes (Bonam et al.; Sonaglioni et al.; Beirag et al.).

There is a big focus on finding epigenomic modalities for treating SARS-CoV-2, as the virus uses the host epigenetic machinery to subdue antiviral components and complete its life cycle within the host (2). Gianella et al. show that severe COVID-19, compared to mild/moderate disease, was characterized by miRNA (non-coding RNAs that regulate gene expression) signatures showing of a profound impairment of innate and adaptive immune responses, inflammation, lung fibrosis, heart failure, and mortality. A combination of high serum miR-22-3p and miR-21-5p, which target antiviral response genes, and low miR-224-5p and miR-155-5p, targeting pro-inflammatory factors, discriminated between severe and mild/moderate COVID-19. Simultaneously, a high leukocyte count and low levels of miR-1-3p, miR-23b-3p, miR-141-3p, miR-155-5p and miR-4433b-5p predicted mortality. Some differentially expressed miRNAs that were modulated directly by SARS-CoV-2 infection in permissive lung epithelial cells could have immense value in defining prognostic biomarkers in stratifying clinical outcomes and preponderance to disease severity for treating the infection earlier.

Given that the different approaches and strategies have been used in designing SARS-CoV-2 vaccines, it is thus important to have comparative analyses between them. To carry out such comparative analyses it is equally important to have high-throughput technologies that can systematically evaluate vaccine attributes in a clinical context, providing gold standards to asses vaccine efficacy. Before the broader deployment of a vaccine, it is crucial to understand the molecular, immunological, genomic, and proteomic bases of the immune responses and their evaluation. This was demonstrated through peptidome analysis of vaccinated individuals by Zhang et al., demonstrating the utility of MALDI-TOF MS in evaluating immune responses after vaccination with CoronaVac along with the discovery of new biomarkers for vaccination and neutralizing antibody generation. Furthermore, Kaznadzey et al., through a simultaneous comparison of Pfizer-BNT162b2, Moderna-mRNA1273, and Sputnik V vaccines, provided the first parallel comparison of immune responses of mRNA and non-mRNA vaccines, with no significant differences after the second challenge in vaccinated individuals who also had COVID-19 before being vaccinated, confirmed by antibodies against the nucleocapsid (N) protein and RBD of SARS-CoV-2 using a Unified ELISA-based assay previously developed in the laboratory. Concurrently, Jochum et al. also demonstrated the value of the high automated throughput Roche Elecsys^®^ Anti-SARS-CoV-2 S assay (referred to as ACOV2S) to detect and quantify the antibody response against the RBD of the S protein in delineating humoral immune responses in mRNA-1273-vaccinated individuals. Therefore, the Elecsys Anti-SARS-CoV-2 S assay (ACOV2S) can be valuable in assessing and quantifying the presence of RBD-directed antibodies against SARS-CoV-2 following vaccination and in the assessment of vaccine-induced humoral immune responses. Similarly, Hosseinian et al. quantified the persistence of humoral immunity (SARS-CoV-2 IgG levels) following vaccination using a coronavirus antigen microarray, which included 10 SARS-CoV-2 antigens. Overall, these aspects have a strong potential to define immune responses in infected and vaccinated individuals and the post-market evaluation of SARS-CoV-2 vaccines in a high throughput manner.

In the context of an effective vaccine, it is also important to identify how the vaccines generate immune response with and without prior exposure to SARS-CoV-2. This knowledge is also valuable in defining the relevance of booster regimens, which have been arbitrarily introduced without a proper definition of timing and consideration of circulating strain at the time. Kaznadzey et al. further showed that vaccinated individuals with Sputnik V with prior SARS-CoV-2 infection showed high levels of antibodies with the ability to effectively neutralize the interaction of RBD with ACE2 following the first dose of Sputnik V, which was consistent with Moderna and Pfizer vaccines, suggesting that anti-RBD signals were comparable among the three vaccines (3, 4). What value do booster doses have in the context of naive and previously infected SARS-CoV-2 individuals? This study also shows that a single administration of Sputnik V (Sputnik Light) could be a sufficient boost to the immune system for those who have had a prior exposure to SARS-CoV-2 infection, but this was not the case with naïve patients who had a two-dose regime, which is in line with other studies on Sputnik Light (6). This, along with the study by Ogric et al., which assessed the humoral immune response after the first, second, and third (booster) doses of BNT162b2 vaccine in SARS-CoV-2 naïve and previously infected healthcare professionals, showed no efficacy of booster shots in individuals with prior exposure to SARS-CoV-2, which is highly relevant in making clinical decisions on booster regimens. A concurrent study by Busa et al., using the same BNT162b2 vaccine, found a potential benefit of the third dose of mRNA vaccine on the lifespan of memory B and T cells, suggesting that booster doses could increase protection against SARS-CoV-2 infection. In contrast, Seidel et al. used another approach in assessing the value of booster using heterologous ChAdOx1 nCoV-19 BNT162b2 prime-boost vaccination in young adults and showed that booster after heterologous vaccination results in adequate immune maturation and potent protection against the Omicron BA.1 variant in young adults, concurring with a study by Dowell et al., which analyzed antibody and cellular responses in adolescents who received COVID-19 vaccination with either ChAdOx1 or an mRNA vaccine (mRNA-1273, BNT162b2). Together, these studies provide a platform for vigorous and tantalizing discussion on the actual value of booster shots in naïve and previously-infected individuals, and also in the context of newly emerging SARS-CoV-2 variants as Omicron can breach immune protection in individuals with weak antibody response despite boosting, as shown by Mohlendick et al., which also underscores the establishment of thresholds for SARS-CoV-2 IgG antibody levels identifying “non”-, “low” and “high”-responders that can be used as an indication for re-vaccination.

Ideally, a robust vaccine must serve to all but that is not always the case, and difficult to achieve. Indeed, we have learnt that the immune response and efficacy of current SARS-CoV-2 vaccines is often conditioned by sex and age (5). Shen et al., who analyzed the innate immune responses to the ChAdOx1 nCoV-19 (AZD1222) adeno-virus-based vaccine in the 25-35, and 60-70-years old age groups, showed that the innate immune response after the first vaccination correlated with neutralizing antibody production and that older people displayed weakened innate immune responses by TLR stimulation and weak or delayed innate immune activation profiles after vaccination compared to young people. Thus, age is an important consideration for vaccine design and efficacy because aging is associated with alterations in the number and quality of innate and adaptive immune cells and mounting of immune responses to immune stimulation. This process is termed immune senescence (6; Pietrobon et al.), which is accompanied by reduced chemotaxis, defective cytokine production, and poor TLR signalling (7), thereby affecting and impairing antigen processing and presentation to T cells and activation of B cells, hence weakening adaptive immunity in older age groups. This was the reason, which could be attributed to the high mortality with SARS-CoV-2 infection observed in the older age group with SARS-CoV-2 infection during the earlier part of COVID-19 pandemic, and subsequently.

In case of Influenza, we have already seen that age is associated with decrease in TLR function in human dendritic cells and with poor antibody response to influenza immunization, further underpinning the importance immune-senescence of the innate immune system in vaccine response as a result of aging (8); and, that immuno-senescence can lead to no or suboptimal response to vaccination, posing a potential risk of breakthrough infection with newly emerging SARS-CoV2 variants. Lopera et al. underpins this aspect and show that the decaying of serum neutralizing activity, both over time and across SARS-CoV-2 variants (using the Pfizer-BioNTechvaccine), is more significant in older men- an essential attribute in vaccine considerations in addition to approaches that can boost the immune response in the elderly, either by adding potent adjuvants or by bringing changes to the route of vaccine administration, augmenting the dose of immunogen, and altering the vaccine design and compositions, allowing more immunogenic targets, must be made (9; Pereira et al.). The current trend in vaccine design seen in most COVID-19 vaccines is the antigenic simplicity, aimed to focus the immune response in those antigens that are likely to induce protective responses. In this context, Pratesi et al took a step further showing that 72 aa long-peptide from SARS-CoV-2 spike protein corresponding to the receptor binding motif (RBM436-507) could generate neutralizing antibodies in mice and was recognized by sera from humans exposed to the infection. Hence, this peptide could be used rather than the entire Spike protein in next-generation of COVID-19 vaccines as an immunogen. This also makes a case for identifying immunogenic peptides from regions other than the spike protein, which can be incorporated in future multi-subunit-vaccines for a broader immune response.

In summary, this special issue encapsulates and provides vigorous discussions on the current and evolving state of SARS-CoV-2 vaccines, their design (Pratesi et al.), and development (Prasad et al.) in the context of innovative strategies that will provide better immune protection, robust responses, variant-specificity, broader coverage, better evaluation and assessment, and improvement in the effectiveness of vaccines in the general population and the elderly.

Together, these articles highlight current and future challenges, and possible strategies to overcome them. Because all SARS-CoV-2 vaccines were rolled out due to urgency, there is a urgent need for an increased understanding of the level of immune response variability; adjuvant in boosting effectiveness; the mechanistic basis, genomic, and proteomic bases of mRNA and non-mRNA vaccines; strain specificity; live attenuated viral vaccine development for better durability; and mode of action of the current and future vaccines that can also integrate how vaccines can be made effective when dealing with factors including age, sex, and health status. Live-attenuated vaccines (LAV), which have been used for targeting measles, Mycobacterium tuberculosis, and polio to induce innate protective immunity, have not been tested for SARS-CoV-2 because of the risks involved, coupled with the paucity of immunological knowledge. LAVs offer better and broader protection against viruses. It may be essential to bend the pandemic curve by providing better therapeutic outcomes through training of the innate immune system.

## Author contributions

All authors contributed to the article and approved the submitted version.
